# Biosynthesized ZnO Nanoparticles Using *Albizia lebbeck* Extract Induced Biochemical and Morphological Alterations in Wistar Rats

**DOI:** 10.3390/molecules26133864

**Published:** 2021-06-24

**Authors:** Doga Kavaz, Amina Lawan Abubakar, Nahit Rizaner, Huzaifa Umar

**Affiliations:** 1 Bioenginering Department, Faculty of Engineering, Cyprus International University, Via Mersin 10, Nicosia 98258, Northern Cyprus, Turkey; suhay1994@gmail.com (A.L.A.); nrizaner@ciu.edu.tr (N.R.); humar@ciu.edu.tr (H.U.); 2 Biotechnology Research Centre, Cyprus International University, Via Mersin 10, Nicosia 99258, Northern Cyprus, Turkey; 3 Department of Biochemistry, Kano State University of Science and Technology, Wudil, Kano P.M.B 3244, Nigeria

**Keywords:** biomedical applications, stabilizing agent, kidney, heart, liver, protein

## Abstract

Nano-based particles synthesized via green routes have a particular structure that is useful in biomedical applications as they provide cheap, eco-friendly, and non-toxic nanoparticles. In the present study, we reported the effect of various concentrations of Zinc oxide nanoparticles synthesized using *A. lebbeck* stem bark extract (ZnO NPsAL) as stabilizing agent on rat biochemical profiles and tissue morphology. Adult Wistar rats weighing 170 ± 5 g were randomly classified into eight groups of five rats each; Group A served as a control fed with normal diet and water. Groups B1, B2, C1, C2, D1, D2, and E were treated with 40 mg/kg and 80 mg/kg of the 0.01, 0.05, and 0.1 M biosynthesized ZnO NPsAL and zinc nitrate daily by the gavage method, respectively. The rats were anesthetized 24 h after the last treatment, blood samples, kidney, heart, and liver tissues were collected for biochemical and histopathological analysis. The rats mean body weight, serum alkaline phosphatase, alanine aminotransferase, creatinine, urea, bilirubin, protein, albumin, globulin, total cholesterol, triacylglycerol, and high-density lipoprotein were significantly altered with an increased concentration of biosynthesized ZnO NPsAL when compared with the control group (*p* < 0.05; *n* ≥ 5). Furthermore, histopathological analysis of treated rats’ kidney, heart, and liver tissue revealed vascular congestion, tubular necrosis, inflammation, and cytoplasmic vacuolation. Biosynthesized ZnO NPsAL showed significant alteration in biochemical parameters and tissue morphology in rats with increasing concentrations of the nanoparticles.

## 1. Introduction

Nanotechnology is an advanced area of studies that utilizes environmentally friendly technologies to synthesize nanoparticles, which are chemically stable, non-toxic, biocompatible, and can be used for biomedical applications [1]. Nanomaterials are smaller-sized particles in the nanoscale dimension with good thermal conductivity, improved catalytic reactivity, and chemical steadiness because of the large surface area [2]. Metal nanoparticles are toxic, difficult to synthesize, expensive, stabilizing agents used in synthesis, and naturally antagonistic [3]. Nano-based particles via green routes have particular structure-specific usefulness in biomedical applications as they provide cheap, eco-friendly, and non-toxic nanoparticles [4]. The surface charge present on nanoparticles contributes to their absorption. Studies revealed ZnO NPs with positively charged ions to be less toxic due to their lower absorption efficiency than those with negatively charged ions [5]. Zinc oxide nanoparticles (ZnO NPs) also have all the properties exhibited by all nanoparticles. It could be used in electronics, biosensors, cosmetics, environmental protection, biology, and the medicinal industry (Rasmussen et al., 2010). ZnO NPs are characterized by their photo-oxidizing and photocatalytic activity against biological and chemical species [3] In addition, ZnO was recognized by the US Food and Drug Administration (FDA) as a generally safe and recognized substance [5].

Hence, ZnO NPs are attractive to many researchers because zinc is the most crucial trace metal found in the human body after iron. It activates many enzymes in the body and regulates blood sugar levels and diverse metabolic activities [6]. Furthermore, diseases such as chronic liver diseases and malabsorption syndrome happened due to homeostasis deviation and zinc deficiency [7]. Synthesis of ZnO NPs via green routes is one of the most straightforward methods, cost-effective, and viable with novel properties than other biosynthesized metal nanoparticles [8]. Some leaves and stem bark extracts used in the synthesis of ZnO NPs include *Costus pictus D. Don* [9], *Parthenium hysterophorus* L. [10], and *A. lebbeck* [11]. Studies report bio-extract-mediated ZnO nanoparticles with exclusive properties revealed novel alternatives to cancer therapy, anti-diabetic, antioxidant, and anti-microbial activity [12,13].

*Albizia lebbeck* is a fast-growing deciduous tree with an open, large, spreading crown; it usually reaches 15–20 m high, with exceptional specimens growing up to 30 m high. The straight, cylindrical bole with impressive specimens up to 300 cm can be 50–100 cm in diameter [14] Saponin isolated from *A. lebbeck* stem bark revealed intense cytotoxic activity against human aqueous cell carcinoma (HSC-2 and HSC-3) [15]. Three Flavonoids (Isookanin, geraldone, and 5-deoxyflavone) isolated from the plant methanolic extract bark revealed antioxidant and anti-diabetic activity in vitro [16].

In the present study, we reported the effect of novel Zinc oxide nanoparticles synthesized using *A. lebbeck* stem extract (ZnO NPsAL) as a stabilizing agent in Wistar rats. The impact of the biosynthesized ZnO NPS on rats body weight and metabolic profile such Alkaline Phosphatase (ALP), Alanine Aminotransferase (ALT), Aspartate Aminotransferase (AST), Creatinine (CREA), Urea, Bilirubin (BIL), Total Cholesterol (TC), Triacylglycerols (TG), High-Density Lipoprotein (HDL-C), total Protein, Albumin ALB and Globulin (GLB). Furthermore, Histopathological examinations of the kidney, heart, and liver of the treated and control rats were also carried out.

## 2. Results

### 2.1. Average Weight of the Animals

**Figure 1:**
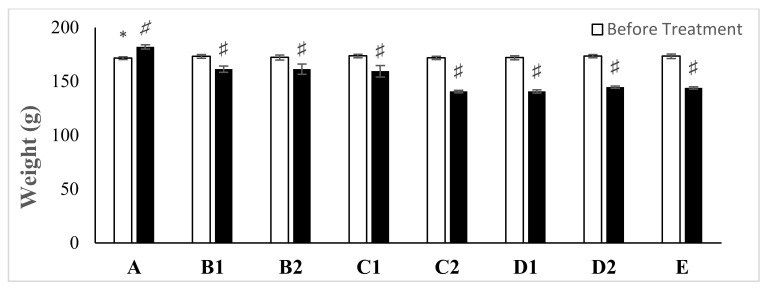
Mean body weight of experimental animals before and after oral administration of various concentrations of ZnO NPs Al. Data are presented as the mean ± SEM of at least *n* ≥ 5 replicate experiments and analyzed using one-way ANOVA followed by Newman–Keuls post hoc analysis. (*) *p* < 0.05; (^♯^) *p* < 0.05. Abbreviations: A, Control; B1, 0.01 M ZnO NPsAL (80 mg kg^−1^); B2, 0.01 M ZnO NPsAL (40 mg kg^−1^); C1, 0.05 M ZnO NPsAL (80 mg kg^−1^); C2, 0.05 M ZnO NPsAL (40 mg kg^−1^); D1, 0.1 M ZnO NPsAL (80 mg kg^−1^); D2, 0.1 M ZnO NPsAL (40 mg kg^−1^); E, Zinc nitrate solution (80/40 mg kg^−1^).

The effect of various concentrations of ZnO NPs synthesized using *A. lebbeck* stem bark aqueous extract was studied on the average weight of experimental animals after 28 days of oral administration (Figure 1). The average body weights of all the experimental groups were quite similar at the initial stage of the experiments. Following 28 days of exposure to synthesized ZnO NPs, the weight of all the concentrations (B1-D2) administered to the animals significantly decreased with increased concentrations when compared with the control groups (*p* < 0.05; *n* ≥ 5; Figure 1). The mean body weight of the control group increased by 10% after the 28 days treatment period (Figure 1), and no harmful sign or mortality was observed in all the groups.

### 2.2. Biochemical Parameters Analysis

The effects of various concentrations of synthesized ZnO NPsAL on serum level markers of liver function were reported in Table 1. The level of ALP decreased with increased concentrations of the synthesized zinc oxide nanoparticles and 0.1M ZnO NPsAL (40 mg kg^−1^ and 80 mg kg^−1^) and significantly decreased ALP level when compared with the control group (*p* < 0.05; *n* ≥ 5; Table 1). The ALT level increased with increased ZnO NPsAL concentrations, and all the concentrations revealed significant differences compared with the control group (*p* < 0.05; *n* ≥ 5; Table 1). Furthermore, no significant difference was observed in all the concentrations of ZnO NPsAL on AST (*p* > 0.05; *n* ≥ 5; Table 1).

**Figure 2:**
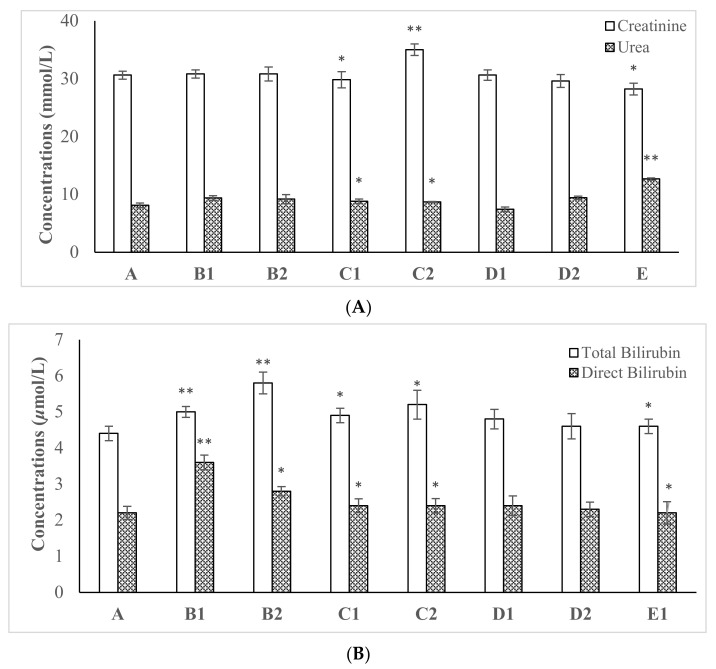
The effect of various concentrations of biosynthesized ZnO NPsAL on serum concentrations in rats (**A**) creatinine and urea (**B**) total bilirubin and direct bilirubin. Data are presented as mean ± SEM of at *n* ≥ 5 least replicate experiments and analyzed using one-way ANOVA followed by Newman–Keuls post hoc analysis. (*) *p* < 0.05; (**) *p* < 0.01; Abbreviations: A, Control; B1, 0.01M ZnO NPsAL (80 mg kg^−1^); B2, 0.01 M ZnO NPsAL (40 mg kg^−1^); C1, 0.05 M ZnO NPsAL (80 mg kg^−1^); C2, 0.05 M ZnO NPsAL (40 mg kg^−1^); D1, 0.1 M ZnO NPsAL (80 mg kg^−1^); D2, 0.1 M ZnO NPsAL (40 mg kg^−1^); E, Zinc nitrate solution (80/40 mg kg^−1^).

Effects of various concentrations of synthesized ZnO NPsAL on serum creatinine, urea, total bilirubin, and direct bilirubin were determined (Figure 2A,B) following 28 days of oral exposure. Oral administration of synthesized ZnO NPsAL altered serum creatinine and urea level in rats, and 0.05 M ZnO NPsAL revealed significant serum creatinine levels relative to the control (*p* < 0.05; *n* ≥ 5; Figure 2A). In addition, zinc nitrate solution (40 mg kg^−1^ and 80 mg kg^−1^) significantly decreased creatinine and urea serum level concentrations (*p* < 0.05; *n* ≥ 5; Figure 2A). Furthermore, serum total and direct bilirubin level significantly increased with an increase in the concentration of the particles, 0.01 M and 0.05 M revealed a significant difference when compared with the control group, whereas 0.1 M did not reveal any significant difference when compared with the control group (*p* > 0.05; *n* ≥ 5; Figure 2B).

**Figure 3:**
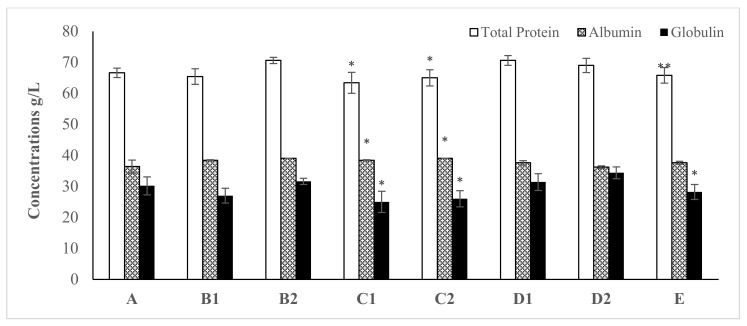
The effect of various concentrations of biosynthesized ZnO NPsAL on total protein albumin and globulin concentrations in rats. Data are presented as the mean ± SEM of at least *n* ≥ 5 replicate experiments and analyzed using one-way ANOVA followed by Newman–Keuls post hoc analysis. (*) *p* < 0.05; (**) *p* < 0.01. Abbreviations: A, Control; B1, 0.01 M ZnO NPsAL (80 mg kg^−1^); B2, 0.01 M ZnO NPsAL (40 mg kg^−1^); C1, 0.05 M ZnO NPsAL (80 mg kg^−1^); C2, 0.05 M ZnO NPsAL (40 mg kg^−1^); D1, 0.1 M ZnO NPsAL (80 mg kg^−1^); D2, 0.1 M ZnO NPsAL (40 mg kg^−1^); E, Zinc nitrate solution (80/40 mg kg^−1^).

The effect of various concentrations of synthesized ZnO NPsAL on rat serum protein, albumin, and globulin (Figure 3; *n* ≥ 5) following 28 days oral exposure was not significant in all concentrations and zinc nitrate solution except 0.05 M ZnO NPsAL when compared with the control (*p* < 0.05; *n* ≥ 5; Figure 3). In contrast, the serum level of total protein and globulin was elevated with all the concentrations compared with the control group (Figure 3; *n* ≥ 5).

**Figure 4:**
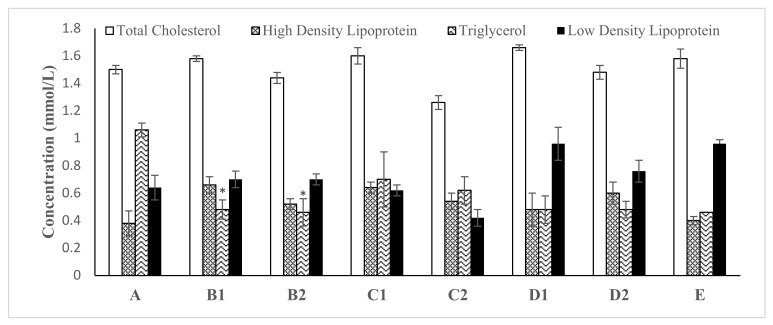
The effect of various concentrations of biosynthesized ZnO NPsAL on TC, HDL, TG, and LDL concentrations in rats. Data are presented as the mean ± SEM of at least *n* ≥ 5 replicate experiments and analyzed using one-way ANOVA followed by Newman–Keuls post hoc analysis. (*) *p* < 0.05; Abbreviations: A, Control; B1, 0.01 M ZnO NPsAL (80 mg kg^−1^); B2, 0.01 M ZnO NPsAL (40 mg kg^−1^); C1, 0.05 M ZnO NPsAL (80 mg kg^−1^); C2, 0.05 M ZnO NPsAL (40 mg kg^−1^); D1, 0.1 M ZnO NPsAL (80 mg kg^−1^); D2, 0.1 M ZnO NPsAL (40 mg kg^−1^); E, Zinc nitrate solution (80/40 mg kg^−1^).

Synthesized ZnO NPsAL changed the rat lipid profiles following 28 days of oral exposure (Figure 4). The total cholesterol level increased in all concentrations at 40 mg kg^−1^ and decreased at 80 mg kg^−1^ as compared with the control group. Furthermore, HDL, TG, and LDL were also significantly increased in all treated groups.

### 2.3. Histopathological Examinations

**Figure 5:**
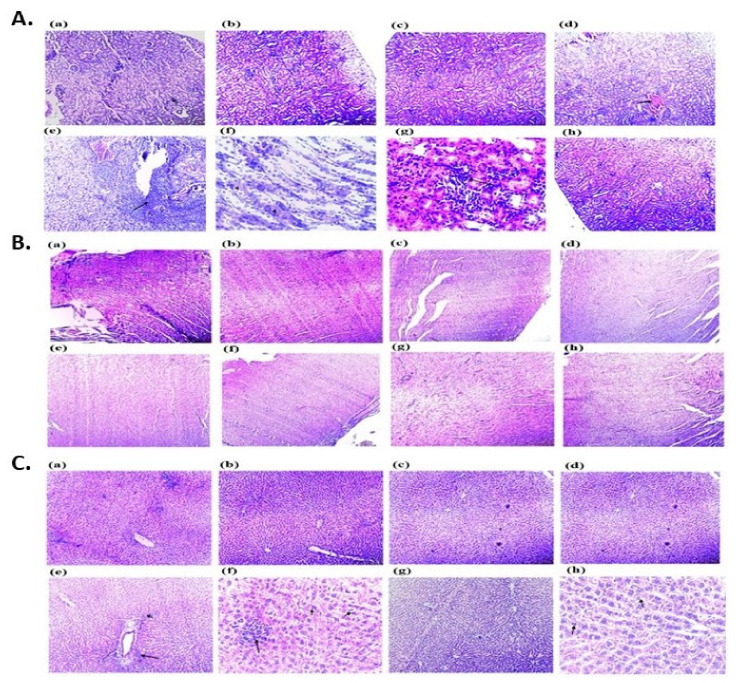
Photomicrographs of; (**A**). Kidney (**B**). Heart (**C**). Liver, section of rats from different groups following 28 days repeated oral exposure to biosynthesized ZnO NPsAL. (**a**) Control group (**b**) 0.01 M ZnO NPsAL (80 mg kg^−1^) (**c**) 0.01 M ZnO NPsAL (40 mg kg^−1^) (**d**) 0.05 M ZnO NPsAL (80 mg kg^−1^) (**e**) 0.05 M ZnO NPsAL (40 mg kg^−1^) (**f**) 0.1 M ZnO NPsAL (80 mg kg^−1^) (**g**) 0.1 M ZnO NPsAL (40 mg kg^−1^) (**h**) Zinc nitrate solution with different concentrations at 0.01 M, 0.05 M and 0.1 M. (Hematoxylin and eosin stain × 100). Abbreviations: ZnO NPsAL; Zinc oxide nanoparticles.

Histopathological investigations of rat renal, cardiac, and hepatic tissue following 28 days repeated oral exposure to various concentrations of biosynthesized ZnO NPsAL. The photomicrograph of rats renal tissue treated with 40 and 80 mg kg^−1^ of 0.05 and 0.1 M ZnO NPsAL showed vascular congestion, inflammation, necrosis, and desquamation of renal epithelium, whereas the control, 0.01 M ZnO NPsAL and Zn(NO_3_)_2_ groups revealed intact tissue morphology without any cellular or tissue damage (Figure 5a; Table 2). Intact cardiac tissue morphology was observed in all the groups treated with various concentrations of biosynthesized ZnO NPs and the control (Figure 5b; Table 2). Furthermore, significant morphological changes such as Cytoplasmic vacuolation and Periportal inflammation were observed in hepatic tissue of rats treated with 40 and 80 mg kg^−1^ of 0.01, 0.05, and 0.1 M ZnO NPsAL (Figure 5c; Table 2). The Zn(NO_3_)_2_ and the control group revealed intact tissue morphology without any cellular or tissue damage (Figure 5).

## 3. Discussion

Researchers have increasingly interested in biologically synthesized metal-oxide nanostructures, especially biosynthesized ZnO NPs, due to their high reactivity, high surface volume, effective interaction with cell membranes, and controlled fabrication with cheap and harmless materials [17]. However, understanding the effect of ZnO NPs on biochemical indices and mechanism of action in the biological system still remain insufficient and is of great concern [18]. Therefore, the present study evaluated the effect of biosynthesized (ZnO NPsAL) on Wistar rat’s body weight and metabolic profile, and histopathological examinations of the kidney, heart, and liver of the treated and control rats were also reported following 28 constitutive days oral administration.

Biosynthesized ZnO NPsAL oral exposure revealed a significant decrease with increasing concentrations of the nanoparticles on the bodyweight of the rats following 28 days oral exposure (*p* < 0.05; *n* ≥ 5; Figure 4), and it could be as a result of a toxic effect of the nanoparticle that is usually associated with bodyweight alteration [19,20]. Similarly, studies reported significant reductions with increased concentration in the average weights of treated rats relative following 14 and 30 days oral exposure to zinc oxide and Silver/Gold nanoparticles, respectively [21].

The results revealed significant alteration on serum level markers (ALP, AST, and ALT) of liver function in rats following 28 days of oral exposure to various concentrations of biosynthesized ZnO NPs (Table 1). The liver is a vital organ that carried out detoxification, metabolism, xenobiotics extraction, and glucose storage [22]. The ALT, ALP, and AST are the reliable ‘markers’ of liver damage and can thus assess liver tissue necrosis [23,24]. Lee et al. (2014) reported increased serum ALP and ALT levels with increased metallic ZnO NPs concentration, similar to the result we obtained [25]. Similarly, higher doses of ZnO NPs were reported to increase ALP level with increasing concentration, and the increase is most likely due to liver injury inflammation produced by the nanoparticles [26].

Serum creatinine, urea, total bilirubin, and direct bilirubin were altered in rats following 28 days of oral exposure to various concentrations of biosynthesized ZnO NPs (Figure 5). AgNPs decreased serum CREA, Urea, and BIL in streptozocin-induced diabetic rats following 28 days of oral exposure [1]. Similarly, serum urea and BIL alteration were reported following daily oral exposure of Au/Ag NPs for 30 days. The decrease in urea could be a result of a decrease in amino acid degradation by the liver, considering the decrease in the level of rat serum albumin [27]. The study revealed that increased red blood cell hemolysis could cause elevated bilirubin level beyond the hepatic function capacity [20].

**Figure 6:**
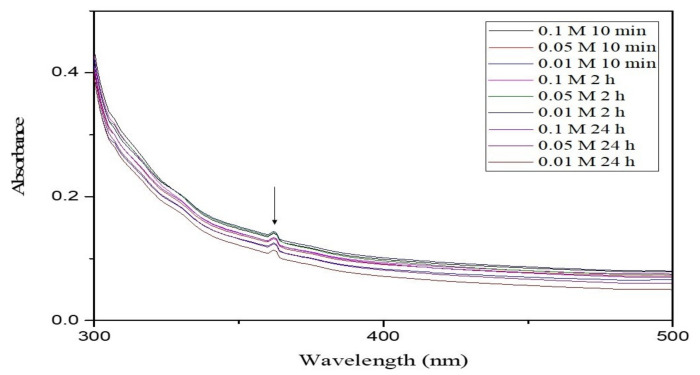
UV–visible spectra of biosynthesized ZnO NPsAL prepared with various concentrations of zinc nitrate at different wavelengths and incubation periods. Adapted from Umar et al. (2019) [19].

**Figure 7:**
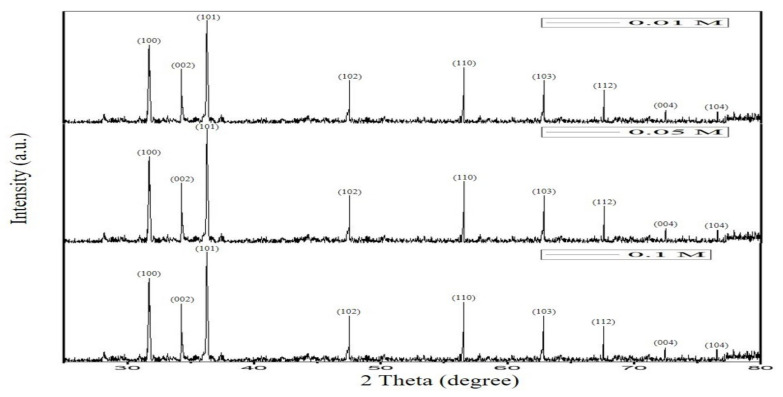
XRD pattern of 0.01M, 0.05M, and 0.1M biosynthesized ZnO NPsAL. Adapted from Umar et al. (2019) [19].

In contrast, rat serum protein levels significantly decrease when treated with 0.05 M ZnNAPsAL, whereas albumin and globulin levels remain normal in all treated groups relative to control (Figure 6). Protein levels decrease significantly in rats with Au/Ag NPs following 30 days of daily oral administration through the gavage method [27]. Similarly, inhalation exposure to ZnO NPs significantly decreased total protein in rats. It could be due to nanoparticle interaction with proteins that interfere with free radicals scavenging enzymes, leading to free radicals and necrosis [28]. Moreover, TC, HDL, TG, and LDL were also significantly increased in all treated groups compared with untreated groups (Figure 7).

Similarly, the study revealed a significant elevation of LDL, TC, and TG in Wistar rat-treated Ag NPs in a concentration-dependent manner [29]. An increase in TG and LDL levels could lead to atherosclerosis and other related cardiovascular disorders [29]. The disparities in TC, HDL, and TG serum levels could be the leading cause of several health diseases due to their essential role in the system [30].

Histopathological examination of rat renal, cardiac, and hepatic tissues following 28 days of repeated oral exposure to various concentrations of biosynthesized ZnO NPsAL showed alteration in the morphology of all the tissues with increased biosynthesized ZnO NPs concentration. Intact morphology was observed in control groups A, B1, B2, and E, and vascular congestion, inflammation, and necrosis were observed in groups C1, C2, D1, and D3. The intact morphology observed in the group treated with zinc nitrate solution and 0.01 M solution might result from fewer Zn ions present in the solution. In addition, morphological alteration observed in the groups treated 0.05M and 0.1M, which might be as a result of the increase in the concentration of Zinc ions present in the solution, and a similar concentration-dependent effect was observed in liver and kidney tissues of rats treated with ZnO nanoparticles [31].

**Figure 8:**
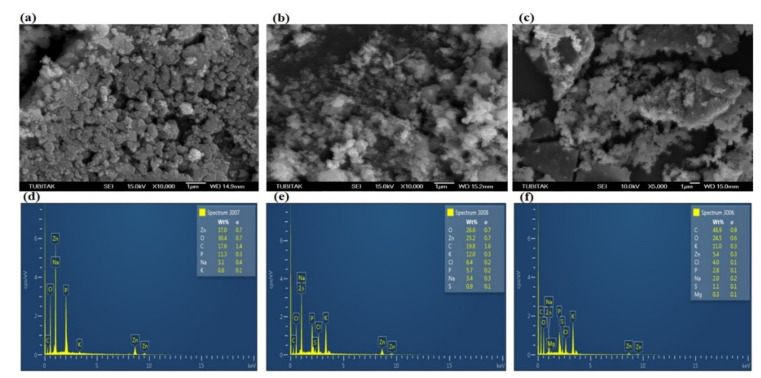
SEM images of biosynthesized ZnO NPsAL: (**a**) 0.1 M, (**b**) 0.05 M, and (**c**) 0.01 M. EDX spectra of the ZnO NPs: (**d**) 0.1 M, (**e**) 0.05 M, and (**f**) 0.01 M. Adapted from Umar et al. (2019) [19].

Histopathological analysis of renal tissue of rats treated with various concentrations of biosynthesized ZnO NPsAL revealed vascular congestion, inflammation, necrosis, and desquamation of renal epithelium (Figure 8a). Glomeruli segmentation, hydropic degeneration in epithelial cells, necrosis, and swelling of epithelial cells were observed in the kidney tissues of mice treated with ZnO NPs [32]. Peritubular inflammation observed in our studies results from progressive chronic kidney diseases like renal tubule interstitial fibrosis, tubular cell differentiation, epithelial–mesenchymal transition, and myofibroblast activation [33,34]. Intact cardiac tissue morphology was observed in all the groups treated with various concentrations of biosynthesized ZnO NPs and the control (Figure 8b).

Similarly, a high concentration of ZnO NPs did not reveal any cellular damage in the cardiac tissue of rats following 30 days of exposure [35]. A study conducted on cardiac tissue of rats exposed to metallic Ag/Au NPs at a dose of 100 mg kg^−1^ for 30 days revealed cellular degeneration and inflammation of the cardiac tissue [27]. Studies showed that NPs induced oxidative tissue damage due to their small sizes and unique properties [36]. Morphological changes were observed in the liver tissues with increased concentration of the NPs as revealed in the liver tissue architecture of the rats, and they might be responsible for inducing apoptosis [37]. A study on the liver tissue morphology in rats treated with ZnO NPs (50 nm) revealed morphological alterations and mitochondria disorders [38]. Similarly, accumulation of ZnO NPs in the hepatic tissue was observed in mice after eight days of exposure to ZnO NPs at the dose of 300 mg kg^−1^ [39,40]. Furthermore, a study on zinc and iron NPs has revealed an adverse morphological effect on rat liver tissue [41].

## 4. Materials and Methods

### 4.1. Chemicals

All chemical and reagents used throughout the experiment were of analytical grade and were supplied by Sigma Aldrich Inc., St. Louis, USA, unless otherwise mentioned. Zinc nitrate, zinc nitrate (Zn(NO_3_)_2_⋅6H_2_O) solutions. Ketamine and xylazine for the anesthesia. The assay kits for Total Cholesterol (TC), Triacyglycerols (TG), High-Density Lipoprotein (HDL-C), Creatinine (CREA), Urea, Bilirubin (BIL), Albumin (ALB), Alkaline Phosphatase (ALP), Alanine Aminotransferase (ALT), and Aspartate Aminotransferase (AST) (Randox Laboratory Limited, Antrim, United Kingdom). Deionized water was used for the preparation of the biosynthesized ZnO NPsAL.

### 4.2. Synthesis of Zinc Oxide Nanoparticles

ZnO NPs using *A. lebbeck* aqueous extract (ZnO NPsAL) was prepared using the method employed by Zare et al. with some minor modifications [42]. Synthesis of ZnO NPs was carried out using 10% 0.1, 0.05 and 0.01 M zinc nitrate (Zn(NO_3_)_2_⋅6H_2_O) solutions and 90% distilled water; then, 10% *A. lebbeck* aqueous extract was added dropwise to the mixture under constant shaking/stirring at a temperature of 60 °C for five consecutive hours so that we can achieve complex formation. After the complex was formed, the mixtures were calcined for 2 h at 350 °C in a muffle furnace.

### 4.3. Characterization of Biosynthesized Zinc Oxide Nanoparticles

**Figure 9:**
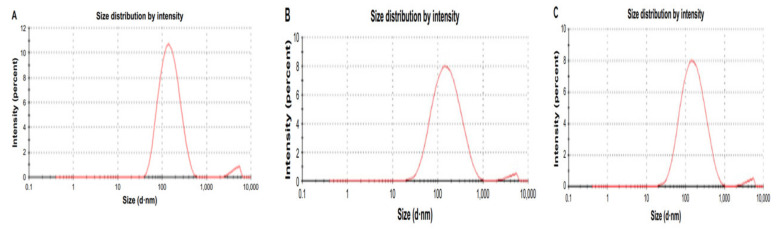
Size of the synthesized ZnO NPs nanoparticle and its distribution (**A**) 0.1 M (**B**) 0.05 M and (**C**) 0.01 M. Hydrodynamic size of the synthesized nanoparticles was analyzed using the Zeta sizer (ZS) analytical instrument (Malvern Nano ZS90), and the results were obtained using ZS290 nano software coupled with the device. Adapted from Umar et al. (2019) [19].

The biosynthesized ZnO NPsAL ZnO NPs were characterized using various spectroscopic and microscopic techniques. A UV-visible spectrophotometer (Shimadzu UV-2450, Duisberg, Germany) was used to evaluate the UV-visible spectrum and recorded between 300 and 800nm (Figure 6). The crystalline structure was analyzed using an X-ray diffractometer (Rigaku ZSX Primus II, Wilmington, MA, USA) (Figure 7). Morphological analysis of the synthesized ZnO NPs coated with platinum was analyzed using a scanning electron microscope (SEM) (JOEL JSM 6335-F, Tokyo, Japan), X-ray spectroscopy (EDS) (Oxford Instruments AZTEC EDS, Osaka, Japan) affixed to the same instrument was used to ascertain the elemental composition (Figure 8). Size of the synthesized nanoparticles was evaluated using the Malvern Zetasizer Nano ZS90 instrument. The average size of the nanoparticles was found to be 66.25 nm with a 0.262 polydispersity index (Figure 9). Uv-Vis, XRD, and SEM-EDS, and Zeta sizer results of biosynthesized ZnO NPsAL were previously reported in our recent studies [10].

### 4.4. Experimental Animals

Albino Wistar rats weighed 170 ± 5 g from the Biological Science Department’s animal house, Bayero University, Kano. The animals were kept in plastic cages in the experimental animal house and allowed to acclimatize for two weeks before the commencement of treatments. Animals were maintained under standard hygienic conditions with alternate 12 h light and dark cycle. Animals were given free access to food and clean water ad libitum. Handling of animals was consistent with relevant guidelines on the care and use of laboratory animals (National Research Council 2011). They were sustained throughout the experimental period in conformity with the Ethics Committee of Bayero University, Kano.

### 4.5. Experimental Design and Treatments

Adult Rats were randomly classified into eight groups of five rats each and they were treated with 0.5ml of the biosynthesized ZnO NPsAL and zinc nitrate daily by gavage method (Sulaiman et al., 2015) for 28 constitutive days as follows: Group A, normal control group, fed with normal diet and water for 28 days; Group B1, administered with 0.01 M biosynthesized ZnO NPsAL (80 mg/kg); Group B2, administered with 0.01 M biosynthesized ZnO NPsAL (40 mg/kg); Group C1, administered with 0.05 M biosynthesized ZnO NPsAL (80 mg/kg); Group C2, administered with 0.05 M biosynthesized ZnO NPsAL (40 mg/kg); Group D1, administered with 0.1 M biosynthesized ZnO NPsAL (80 mg/kg); Group D2, administered with 0.1 M biosynthesized ZnO NPsAL (40 mg/kg); Group E, administered with 10% Zinc nitrate solution (80 mg/kg). The amount of Zinc nitrate solution used has the same concentration as the amount of Zinc nitrate solution used in the synthesis of the nanoparticle.

### 4.6. Blood Sampling and Tissue Preparation

The rats were weighed, anesthetized with 60/6 mg/kg of ketamine/xylazine intraperitoneally 24 h after the last treatment. Blood samples were taken in a clean and sterile sample container. The serum was obtained after the blood sample was centrifuged at 3000 rpm for 15 min stored at −80 °C prior to analysis. The rats were sacrificed and kidney, heart, and liver tissues were separated, weighed, and kept in 10% formaldehyde prior to histopathological examinations.

### 4.7. Biochemical Parameters Analysis

The serum levels of AST, ALT, ALP, ALB, BIL, CREA, urea, serum TC concentration, HDL-C, and TG were assayed using the Randox assay kits (Randox Laboratory Limited County Antrim, United Kingdom) according to the manufacturer’s instructions. The concentration of serum LDL-C was estimated using the Fried Ewald formula [43]. The protein content of the serum was determined using the biuret method as previously described by [44].

### 4.8. Histopathological Examination

Kidney, heart, and liver tissue fragments were removed and fixed with 10% formaldehyde, dehydrated with ascending grade of alcohol, cleared with toluene, infiltrated with molten paraffin wax. The microtone section was stained with the hematoxylin and eosin (H&E) staining technique, mounted in mounting media and examined with a Leica DM 750 microscope, and photographed with Leica 1CC 50 HD Camera.

### 4.9. Statistical Analysis

Data are given as means ± standard errors of the mean (SEMs). Statistical comparisons were carried out using one-way analysis of variance (ANOVA) followed by Newman–Keuls post hoc analysis where necessary on SPSS version 23 software. The significance levels were set up at the 5%, 1%, and 0.1% levels, where *p* < 0.05 was considered significant, *p* < 0.01 insignificant and *p* < 0.0001 considered highly significant.

## 5. Conclusions

The present study demonstrated the effect of various concentrations of ZnO NPs synthesized using *Albizia lebbeck* stem bark extract as a chelating agent on biochemical parameters and tissue morphology in Wistar rats. Biosynthesized ZnO NPs caused a significant alteration in rats’ mean body weight, serum alkaline phosphatase, alanine aminotransferase, creatinine, urea, bilirubin, protein, albumin, globulin, total cholesterol, triacylglycerol, low density, and high-density lipoprotein in a concentration-dependent manner. Additionally, histopathological analysis of treated rats’ kidney, heart, and liver tissue revealed vascular congestion, tubular necrosis, inflammation, and cytoplasmic vacuolation.

## Figures and Tables

**Table 1:** The effect of various concentration of biosynthesized ZnO NPsAL on ALP, ALT, and AST concentrations in rats.

Group	ALP (U/L)		ALT (U/L)		AST (U/L)	
Control	37.60	±4.1	3.80	±0.5	11.20	±0.7
0.01M ZnO NPsAL (80 mg kg^−1^)	37.00	±7.4	6.20	±0.5 *	12.40	±1.1
0.01M ZnO NPsAL (40 mg kg^−1^)	36.40	±2.1	6.00	±0.5 *	11.80	±0.7
0.05M ZnO NPsAL (80 mg kg^−1^)	31.60	±6.8	7.00	±0.9 *	12.40	±1.1
0.05M ZnO NPsAL (40 mg kg^−1^)	33.20	±4.1	6.20	±0.5 *	11.20	±0.7
0.1M ZnO NPsAL (80 mg kg^−1^)	11.80	±3.4 *	6.80	±0.5 *	13.00	±0.9
0.1M ZnO NPsAL (40 mg kg^−1^)	23.40	±6.6 *	6.80	±1.0 *	11.20	±0.7
Zinc nitrate solution	34.20	±6.0	6.20	±0.5 *	10.00	±0.9

Data are presented as the mean ± SEM of at least *n* ≥ 5 replicate experiments and analyzed using one-way ANOVA followed by Newman-Keuls post hoc analysis. (*) *p* < 0.05.

**Table 2:** The summary of histopathological examination of renal, cardiac and hepatic tissues.

Group	Concentration	Kidney	Heart	Liver
A	Control	Intact tissue morphology	Intact tissue morphology	Intact tissue morphology
B1	0.01 M 80 mg kg^−1^	Intact tissue morphology	Intact tissue morphology	Intact tissue morphology
B2	0.01 M 40 mg kg^−1^	Intact tissue morphology	Intact tissue morphology	Intact tissue morphology
C1	0.05 M 80 mg kg^−1^	Vascular congestion	Intact tissue morphology	Cytoplasmic vacuolation
C2	0.05 M 40 mg kg^−1^	Inflammation, necrosis, and desquamation of renal epithelium	Intact tissue morphology	Cytoplasmic vacuolation
D1	0.1 M 80 mg kg^−1^	Peritubular inflammation and necrosis	Intact tissue morphology	Periportal inflammation
D2	0.1 M 40 mg kg^−1^	Renal tubular necrosis	Intact tissue morphology	Periportal inflammation and fibrosis
E	Zn(NO_3_)_2_	Intact tissue morphology	Intact tissue morphology	Intact tissue morphology

## Data Availability

The authors confirm that the data supporting the findings of this study are available within the article.

## Abbreviations

ZnO NPs	zinc oxide nanoparticles
NPs	nanoparticles
ZnO NPsAL	Zinc oxide nanoparticles synthesized using *A. lebbeck* stem extract
FDA	food and drug administration
ALP	alkaline phosphatase
ALT	alanine aminotransferase
AST	aspartate aminotransferase
CREA	creatinine
TBIL	total bilirubin
DBIL	Direct bilirubin
TC	Total Cholesterol
TG	Triacylglycerols
HDL-C	High Density Lipoprotein
ALB	Albumin
GLB	Globulin
Uv-Vis	UV-visible spectrophotometer
XRD	X-ray diffractometer
SEM	scanning electron microscope
EDS	X-ray spectroscopy
A	normal control group
B1	treated with 0.01 M ZnO NPsAL (80 mg kg^−1^)
B2	treated with 0.01 M ZnO NPsAL (40 mg kg^−1^)
C1	treated with 0.05 M ZnO NPsAL (80 mg kg^−1^)
C2	treated with 0.05 M ZnO NPsAL (40 mg kg^−1^)
D1	treated with 0.1 M ZnO NPsAL (80 mg kg^−1^)
D2	treated with 0.1 M ZnO NPsAL (40 mg kg^−1^)
E	treated with Zinc nitrate solution (80/40 mg kg^−1^)
